# Greater flowering and response to flooding in *Lythrum virgatum* than *L. salicaria* (purple loosestrife)

**DOI:** 10.1093/aobpla/plad009

**Published:** 2023-03-01

**Authors:** Kali Z Mattingly, Brenna N Braasch, Stephen M Hovick

**Affiliations:** Department of Evolution, Ecology and Organismal Biology, The Ohio State University, Columbus, OH, USA; Department of Evolution, Ecology and Organismal Biology, The Ohio State University, Columbus, OH, USA; Department of Evolution, Ecology and Organismal Biology, The Ohio State University, Columbus, OH, USA

**Keywords:** Cultivation, invasive species, Lythraceae, ornamental, submersion

## Abstract

Newly introduced trait diversity can spur rapid evolution and facilitate local adaptation in the introduced plant *Lythrum salicaria*. The horticultural plant *L. virgatum* might further introduce meaningful trait variation by escaping into established *L. salicaria* populations or by hybridizing with *L. salicaria*. Although many experiments have focused on *L. salicaria* genotypes, relatively little is known about *L. virgatum* ecology. We used a greenhouse common garden to compare traits and flood response of *L. salicaria* and *L. virgatum* collected from two sources each in their native range. We tested the hypotheses that these two wetland taxa have comparable responses to flooding (inundation), and that flood tolerance correlated to higher fitness. Flooding produced stronger stress responses in *L. virgatum.* Compared to *L. salicaria, L. virgatum* shifted more aboveground allocation away from reproduction, decreased inflorescence biomass by 40% more, and produced 7% more stem aerenchymatous phellum, a specialized tissue that maintains aeration. Despite these more pronounced responses to flooding stress, *L. virgatum* had higher fitness (inflorescence biomass and reproductive allocation) than *L. salicaria*. Overall, *L. virgatum* differed from *L. salicaria* in functionally important ways. *Lythrum virgatum* persisted under flooding and produced more reproductive biomass than *L. salicaria* under both flooded and non-flooded conditions. However, inundation stressed *L. virgatum* more than *L. salicaria. Lythrum virgatum* is likely able to establish into the wetland habitats in which *L. salicaria* prevails but may possess broader habitat tolerances.

## Teaser

We sought to compare the little-studied ornamental European wand loosestrife (Lythrum virgatum) to its invasive relative purple loosestrife (L. salicaria). Some have assumed the two loosestrifes to be indistinguishable, while others have supported European wand loosestrife as a planting alternative to purple loosestrife. In a greenhouse experiment, we compared the two loosestrifes in terms of ecologically meaningful traits. We revealed similarities as well as important differences that challenge previous assumptions about European wand loosestrife. Our findings are relevant in the applied context of purple loosestrife invasions. European wand loosestrife may contribute an underappreciated store of diversity.

## Introduction

High intraspecific variability in introduced plant species can enable rapid evolution in the non-native range. For species that have been introduced repeatedly from multiple sources, high genetic and trait variation within populations is common (Dlugosch and Parker 2008; Dlugosch *et al.* 2015; Hodgins *et al.* 2018), and at the macroscale, such intraspecific trait diversity can stratify across different habitats or even climates (Colautti and Barrett 2013). A diversity of genotypes and phenotypes also provides the raw material for an introduced lineage to evolve in the longer term, with admixture among diverse genotypes creating novel trait combinations (Schierenbeck and Ellstrand 2009; Hovick and Whitney 2014). High diversity and cryptic admixture are especially common in escaped ornamental plants (Culley and Hardiman 2009). Cultivars with desirable traits are often sourced from geographically disparate sources in the native range and selected through breeding. It is thus unsurprising that horticultural escapes are common sources of invasive florae (Reichard and White 2001; Lehan *et al.* 2013; Beaury *et al.* 2021), a conservation problem that horticulturists are beginning to recognize (Schnelle and Gettys 2021).

Purple loosestrife (*Lythrum salicaria*) is a diverse Eurasian perennial introduced to North America both intentionally as an ornamental and unintentionally through ballast water (Stuckey 1980). Seeds rather than asexual propagules facilitate the species’ spread over long distances (Yakimowski *et al.* 2005). Introductions date back to the early 1800s, but *L. salicaria* remained scarce in much of North America until the mid-1900s (Stuckey 1980). Now, *L. salicaria* is considered one of the worst invasive plant species in Eastern North America, with estimates of its damage and management costing up to $60-$80 million annually in the U.S. over the past 25 years (estimates adjusted for inflation; ATTRA 1997, as cited in Pimentel *et al*. 2005; Cusack *et al.* 2009; Diagne *et al.* 2020). The rise of *L. salicaria* has been driven by a variety of factors, including its competitive ability (Gaudet and Keddy 1988; Hovick *et al.* 2011) and function as an ecosystem engineer (Fickbohm and Zhu, 2006), potentially compounded by its release from natural enemies present in the native range (Blossey and Nötzold 1995; Hunt-Joshi *et al.* 2004). Perhaps more important than any of these other processes has been local adaptation (Colautti and Barrett 2013; Wu and Colautti 2022). Introduced *L. salicaria* has adapted across latitudinal gradients (Colautti and Barrett 2013; Wu and Colautti 2022), apparently driven by high genetic and phenotypic diversity in the introduced range (Chun *et al.* 2009; Colautti and Barrett 2011; Shi *et al.* 2018; Middleton *et al.* 2019). Population genetic studies of introduced *L. salicaria* show evidence of sexual reproduction among multiple introductions from European sources (Chun *et al.* 2009; Middleton *et al.* 2019), confirming hypotheses of early botanists who observed the species’ patchwork expansion across North America over the 20^th^ century (Stuckey 1980). Earlier researchers hypothesized that a North American congener, *L. alatum* (winged loosestrife), might contribute through sexual reproduction to the diversity of invasive *L. salicaria* populations (summarized in Galatowitsch *et al.* 1999). However, studies have so far found little evidence of this phenomenon (Strefeler *et al*. 1996 a; Houghton-Thompson *et al.* 2005); this is perhaps unsurprising given that *L. alatum* is uncommon, restricted to high quality prairie fragments.

Another common *Lythrum* could complicate this story. *Lythrum virgatum* (European wand loosestrife) gained popularity as an ornamental perennial in North America (Harp 1967) after governments began regulating the sale of *L. salicaria*. Although often marketed as a sterile and non-invasive alternative to *L. salicaria*, cultivars are not sterile, they are only self-sterile. *Lythrum* must reproduce sexually to achieve high seed set (Colautti *et al*. 2010 b). *Lythrum virgatum* cultivars produce fertile crosses with *L. salicaria* experimentally (Anderson and Ascher 1993; Ottenbreit and Staniforth 1994) and in nature under ambient pollinator activity (Lindgren and Clay 1993; Amon *et al.* 2007). Because they cross readily, many have considered *L. salicaria* and *L. virgatum* the same species (Rendall 1989; Anderson and Ascher 1993; Strefeler *et al.* 1996b; Wilson *et al.* 2004; Anderson 2019). To date, taxonomic studies have not included precise enough techniques to settle the question (Strefeler *et al*. 1996 b; Haining and Graham 2007; Morris 2007). Such assessments are also complicated by confusion surrounding the origin of many cultivars labeled or mislabeled *L. virgatum* (Strefeler *et al*. 1996 b; Jon Peter and David Galbraith, Royal Botanical Gardens Ontario, pers. comm.). Competing interests may also be at stake. Horticulturists benefit from taxonomic splitting because *L. virgatum* is less often regulated than *L. salicaria* (though an increasing number of governments are doing so). Conservationists would benefit from considering them the same species, because any regulations applying to *L. salicaria* would then also apply to *L. virgatum*.

**Figure 1.**
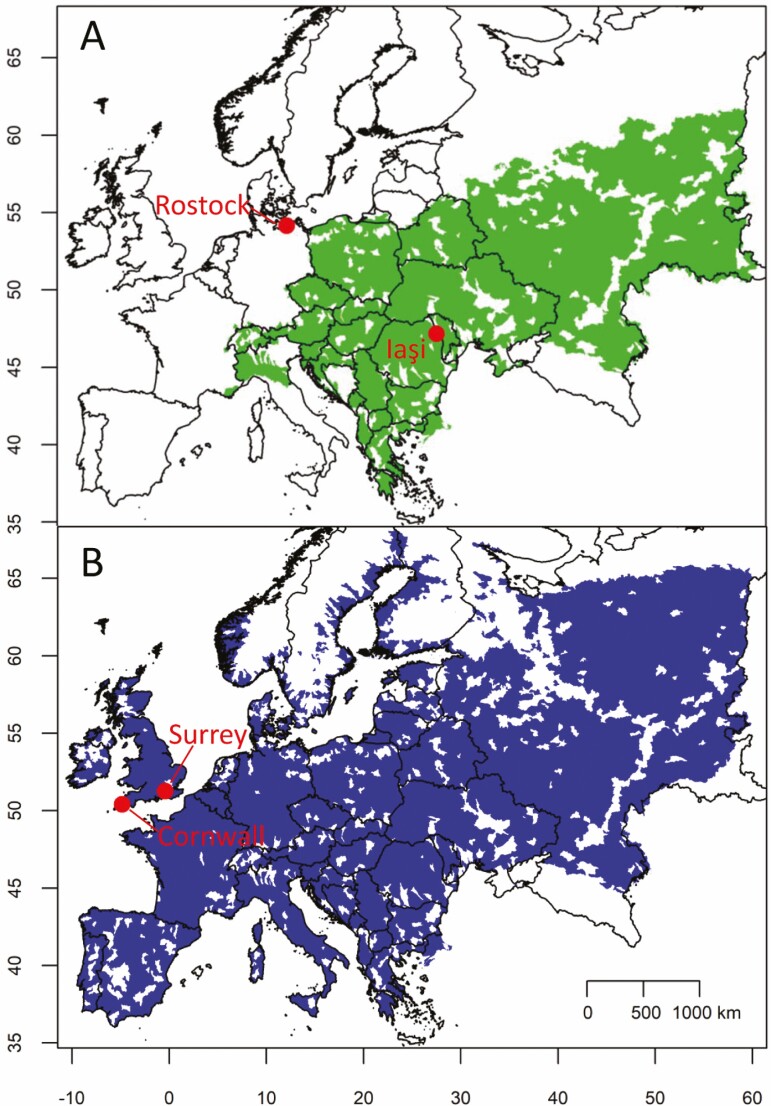
Native European ranges of *Lythrum virgatum* and *L. salicaria* from HydroBASINS BioFresh BioMatrix, which record contemporary distributions across European level 8 watersheds (Lehner and Grill 2013). Points mark sources of accessions used in our greenhouse experiment. (A) *Lythrum virgatum* from Iaşi, Romania, and Rostock, Germany (where the species is naturalized; Webb 1968). (B) *Lythrum salicaria* from Cornwall and Surrey, UK.

Regardless of whether taxonomists determine *L. salicaria* and *L. virgatum* to be the same species, *L. virgatum*’s prevalence in landscaping and in naturalized populations in North America (Wherry *et al.* 1979; USDA, NRCS 2021) represents a unique store of genetic and phenotypic variability. Here we refer to the two taxa as distinct species, while acknowledging the ongoing debate. The native ranges of these *Lythrums* overlap, but *L. salicaria*’s range extends farther west and north than that of *L. virgatum* (Fig. 1). Although North American *L. salicaria* has been introduced from multiple native-range sources (Chun *et al.* 2009; Middleton *et al.* 2019), the sources of popular *L. virgatum* cultivars are unknown (Jon Peter and David Galbraith, Royal Botanical Gardens Ontario, pers. comm.). Native habitats for the two taxa are apparently similar, with *L. virgatum* described as occupying ‘damp places’ and *L. salicaria* ‘damp grasslands, banks’ (Haining and Graham 2007). Ploidy differs in the two species. *Lythrum virgatum* is diploid and mostly *n* = 15 (Graham and Cavalcanti 2001). *Lythrum salicaria* shows more variation in both base chromosome number and ploidy, with tetraploids (*n* = 15) most common in the native range (Graham and Cavalcanti 2001; Kubátová *et al.* 2008) and the only cytotype detected in North America (Kubátová *et al.* 2008). Several morphological traits differ between the two species. *Lythrum virgatum* has traditionally been reported as being overall less robust and having smaller flowers, narrower leaves, shorter height, and less pubescence overall than *L. salicaria* (Haining and Graham 2007).

The trait diversity *L. virgatum* could contribute, through hybridization or establishment into the wild, may be relevant to the evolution and ecology of *L. salicaria,* particularly in the context of invasions. Trait variation in *L. salicaria* facilitates local adaptation in the introduced range (Colautti and Barrett 2013; Wu and Colautti 2022). The most relevant components of such variation are those that translate into ecologically pertinent phenotypes, which may facilitate invasiveness (Dlugosch *et al.* 2015). An important ecological context for these wetland species is flooding. Many researchers have studied effects of flooding on *L. salicaria* from the introduced range (Merendino *et al.* 1990; Haworth-Brockman *et al.* 1993; Weiher *et al.* 1996; Mal *et al.* 1997; Stevens *et al*. 1997, 2002; Lempe *et al.* 2001; Ferrarese and Garono 2011), native range (Bastlová *et al.* 2004), and both (Chun *et al.* 2007; Chun 2011). *Lythrum salicaria* responds to flooding by producing adventitious roots from stems and aerenchymatous phellum on stem and roots (Schenck 1889; Stevens *et al*. 1997, 2002; Lempe *et al.* 2001). Studies of *L. salicaria* have shown these adaptations function to maintain aeration of both root and aboveground cells experiencing inundation (Stevens *et al.* 2002), and, along with other trait shifts (Haworth-Brockman *et al.* 1993; Mal *et al.* 1997; Stevens *et al*. 1997, 2002), facilitate flood tolerance. Flood response of *L. salicaria* has not been compared to that of *L. virgatum*. Assertions that the two taxa are the same species (Rendall 1989; Anderson and Ascher 1993; Strefeler *et al*. 1996 b; Wilson *et al.* 2004; Anderson 2019)—combined with observations that *L. virgatum* produces aerenchyma (Schenck 1889) and grows in wet conditions in the native range (Haining and Graham 2007; Lehner and Grill 2013)—suggest *L. virgatum* and *L. salicaria* should show comparable responses to flooding. Similarities in the two taxa’s responses to moisture gradients would suggest that introduced *L. virgatum* could escape into the same habitats long occupied by *L. salicaria*, potentially introducing new trait diversity. Differences in flood response might indicate some differentiation in the types of sites *L. virgatum* could colonize. We compared traits and flood responses of *L. salicaria* and *L. virgatum* collected from sources in their native range. We used morphological data from a greenhouse experiment to test the hypotheses that: 1) the two species have comparable flooding response, and 2) that flood tolerance is associated with higher fitness (quantified here as inflorescence biomass) under flooded conditions in both species.

## METHODS

### Plant material

**Figure 2.**
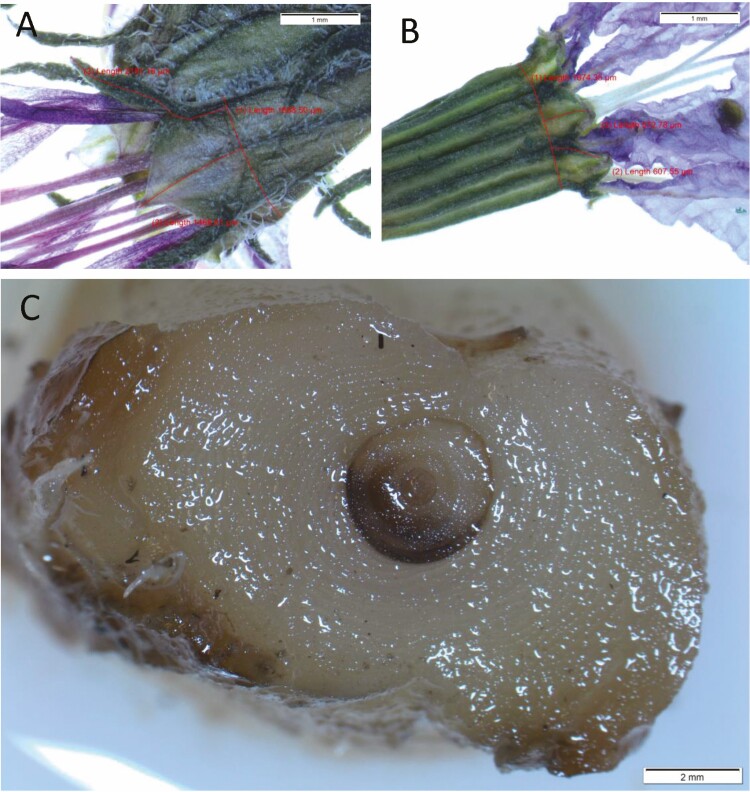
Morphology relevant to our experiment comparing traits and flood response in *Lythrum virgatum* and *L. salicaria,* including identifying characters (Haining and Graham 2007) used to confirm species identities for each accession. Photos and measurements taken with Olympus SZX7, SC100, and cellSens (Olympus, Tokyo, Japan). (A) Collection from Cornwall, UK, consistent with *L. salicaria*: hairy with calyx appendages nearly twice as long as calyx lobes. (B) Collection from Rostock, Germany, consistent with *L. virgatum*: glabrous with calyx appendages shorter than calyx lobes. (C) Example of preserved stem cross section used to measure aerenchyma production, or the ratio of stem aerenchyma area to total stem area (sample from flooded *L. virgatum,* Iaşi, Romania).

We obtained seeds of two different accessions for each species from Europe. All four accessions originally hailed from local natural collections rather than horticultural sources, an important requirement given the unknown origins of many cultivars (Strefeler *et al*. 1996 b). Two accessions of *L. salicaria,* one each from Cornwall and Surrey, UK; were provided by the Millennium Seed Bank Partnership, Kew Gardens. We obtained two *L. virgatum* accessions, one from Rostock Botanical Garden, Rostock, Germany, and one from Anastasie Fatu Botanic Garden, Iaşi, Romania. Purported species identities of each accession matched identifying traits described by Haining and Graham (2007). Compared to the *L. salicaria* accessions, *L. virgatum* plants were overall smaller with smaller flowers (note flower size differences in Fig. 2A,B) and were glabrous rather than hairy (Fig. 2A,B). We also measured calyx appendages and calyx lobes; *L. salicaria* has long appendages, while those of *L. virgatum* are much shorter (Haining and Graham 2007). Ratios of calyx appendage length to calyx lobe length (Fig. 2A,B) aligned with expectations: *L. salicaria* Cornwall, UK = 1.988 [1.373—2.529], *L. salicaria* Surrey, UK = 1.891 [1.165—3.274], *L. virgatum* Germany = 0.662 [0.320—1.206], *L. virgatum* Romania = 0.914 [0.530—1.211] (means and ranges across 3—6 flowers from 4—6 individuals of each accession).

### Experimental design

Seeds from all accessions had high survivability (~90%) when germinated in petri dishes on moist filter paper. We transplanted germinants into fertilizer-free propagation mix (LM-AP All Purpose Mix, Lambert, Rivère-Ouelle, Quebec, Canada). We retained four robust genets of each of the four accessions to serve as maternal plants for our experiment. Within accessions, these genets were presumably distinct genotypes, although no information was provided regarding how our source seeds were originally collected. We grew and acclimated genets for about one year in a greenhouse at The Ohio State University (Columbus, Ohio, USA) under 16 h/d of light. One *L. salicaria* (from Cornwall, UK) died during this time, bringing the total to 15 genets. We cloned genets by planting stem cuttings dipped in rooting hormone (Garden Safe TakeRoot, Spectrum Brands, Middleton, Wisconsin, USA) in germination trays. We allowed cuttings at least 20 d to establish, then selected robust individuals to be transplanted into 20 cm diameter × 13 cm tall pots with drainage holes, filled with propagation mix (LM-ORG, Lambert, Rivière-Ouelle, Québec, Canada) mixed with ~1% solution of dish detergent (Ajax, Colgate-Palmolive, New York, New York, USA) to aid rewetting. Prior to initiation of treatments, these pots were kept in trays and bottom-watered every other day by filling the tray to a depth of ~5 cm (8 cm below the top of the pot).

We used a randomized block design with 64 individuals total: two greenhouse rooms × two species × two seed accessions × four genets (with the contribution of one *salicaria* Cornwall mother doubled) × two experimental treatments. This procedure resulted in eight replicates per species (two accessions × four clones) per treatment in each of two blocks (i.e., greenhouse room). We randomized placement of individuals within replicates.

### Treatments—

After a period of 60 d post-transplant, we began preparing plants for the start of our two treatments: flooded and non-flooded conditions. We first spread a ~3 cm layer of coarse sand (Paver Base, Quikrete, Atlanta, Georgia, USA) across the top of all pots to prevent flooded individuals from floating. All individuals, flooded and non-flooded, received sand to control for any effect this media addition might introduce. We trimmed aboveground biomass to standardize individuals to a similar size prior to the start of treatments. We retained the single largest stem, trimming any additional stems flush to the sand. We trimmed the single retained stem to a height of 30 cm above the sand and removed any lateral branches > 1 cm long. We collected the removed plant material, separated it into vegetative and inflorescence fractions, measured the dry mass (after drying at 60°C), and used these data as model covariates to control for pre-experiment size differences among individuals. We also noted at this time which individuals were flowering prior the start of the experiment. We then nested all 13 cm tall pots within larger ~19 L pots. For the flooded treatment, the outer pots were 28 cm in height and did not contain drainage holes. For the non-flooded controls, the outer pots were holed pots with heights of 24–31 cm. These heights varied due to product availability, but we spread pots of different heights evenly across different replicates and accessions. Nine days after these preparations were completed, we initiated the flooding treatment. For flooded individuals, we lined the unholed outside pots with 2 mm contractor bags (Up & Up, Target, Minneapolis, Minnesota, USA) to ensure they were watertight and filled them to a water depth of ~27 cm (14 cm above the top of the inner pot containing the plant). An inundation depth of 14 cm is near the upper limit reported for *L. salicaria* in the wild, and the species is seen across a wide range of moisture regimes, from irregular soil saturation to permanent inundation (NCHRP 1996). For non-flooded individuals, bottom-watering proceeded as previously described. We topped off water every other day and replaced flooded individuals’ water every 7 d to limit algae growth. We ran the experiment for a total of 53 d, at which point some flooded individuals were showing signs of senescence. Given these species’ invasive tendencies in the area we conducted this study, we did not cross-pollinate individuals, meaning they did not set seed (these heterostylous species require crossing to set seed [Colautti *et al*. 2010 b]).

### Data collection

To quantify each species’ response to flooding, we collected aboveground biomass and sampled stem aerenchymatous phellem at the end of the experiment. We separated the aboveground material into vegetative and inflorescence biomass and measured dry masses. Fractioning the biomass allowed us to examine potential differences in allocation strategies, which have been shown to be genetically controlled in *L. salicaria* (Olsson 2004; Colautti and Barrett 2011). We investigated total aboveground biomass as a metric of overall plant size and vigor, as well as two indicators of fitness. The first was inflorescence biomass, to gauge total reproductive output. The other fitness metric was the ratio of inflorescence biomass to total aboveground biomass, or an individuals’ reproductive allocation. We also quantified the aerenchymatous phellum that formed around the interior vascular cylinder of the stem (Fig. 2C)—a flood tolerance response well-characterized in *L. salicaria* (Schenck 1889; Stevens *et al*. 1997, 2002; Lempe *et al.* 2001) and also observed for *L. virgatum* (Schenck 1889). From the largest stem of each individual, we cut a 4 cm portion starting 1 cm from the top of the soil and submerged it in FAA (Formalin-Alcohol-Acetic Acid, 10%: 50%: 5% + 35% water). Then, we hardened the tissue by storing it in 70% ethanol. From each stem section, we used a razor blade to take a cross section from the middle (~3 cm from the top of the soil). We photographed cross sections (Olympus SZX7, SC100, and cellSens, Olympus, Tokyo, Japan) and used winFOLIA (Regent Instruments, Sainte-Foy, Quebec, Canada) to draw measurement polygons around the inner vascular cylinder and the outer aerenchyma. The aerenchyma area equaled the area inside the outer polygon minus that inside the inner polygon. We calculated aerenchyma production: the ratio of aerenchyma area to total stem area. Aerenchyma measurements excluded data from one flooded German *L. virgatum* individual that died a few days before harvest and for which the aerenchyma had become visibly deflated. Our dataset is available as Supporting Information 1.

### Data analysis

We tested Hypothesis 1 by comparing all traits across species by treatments and Hypothesis 2 by testing whether our two fitness indicators (inflorescence biomass and reproductive allocation) were predicted by our flood tolerance metric (aerenchyma production). To compare how many individuals of each species were flowering prior to the start of the experiment, we used a Pearson’s Χ^2^ test (stats::chisq.test; R v. 4.0.3, R Development Core Team 2020). For all other analyses, we used linear mixed models with general purpose optimization (Pinheiro *et al.* 2020). For Hyp. 1 models, we tested whether trait values were predicted by species × treatment interactions. Hypothesis 2 models had a fitness metric as a response variable and aerenchyma production as a predictor. Species was also included as a predictor in Hyp. 2 models, because the relationship between aerenchyma production and fitness might be moderated by species. Hypothesis 2 models included only individuals that produced aerenchyma (flooded treatment). All Hyp. 1 and 2 linear mixed models included random effects for block (i.e., replicate) and accession, and we weighted variance by accession to address variance heterogeneity. Analyses of the three biomass-based responses included as covariates their respective pretreatment measurements. These covariates accounted for the fact that some plants had grown larger than others prior to being trimmed to a standard size at the start of the experiment, and some had initiated flowering while others had not. Analyses of traits other than total aboveground biomass also included that trait as a covariate, so that model results could be interpreted independent of potential size-based trait differences. The statistics we present for each predictor from the linear mixed models are P-values for significance, calculated from type III sums of squares (Fox and Weisberg 2019), and β coefficients to describe the strength of relationships. For continuous predictors, β coefficients are interpreted as regression coefficients or slopes of the relationship of that predictor to the response (e.g., for Hyp. 2, the relationship of aerenchyma production to a fitness metric). For categorical predictors, β coefficients contrast means of each category (e.g., for Hyp. 1, the mean trait value for *L. virgatum* is compared to the mean for *L. salicaria*).

## RESULTS

**Figure 3.**
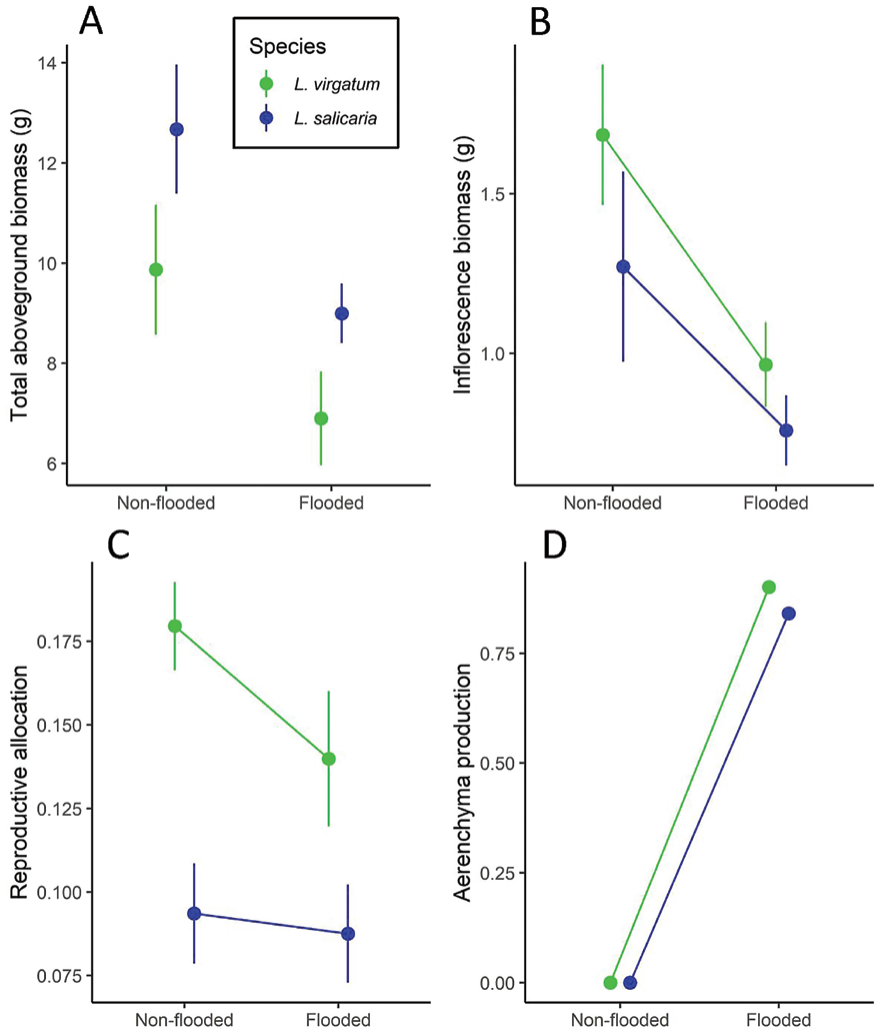
Comparisons of raw trait means (± standard error) for non-flooded and flooded individuals of *Lythrum virgatum* and *L. salicaria*. Connecting lines added only in cases where analyses indicated a significant species × treatment interaction (Table 1). (A) Total aboveground biomass. (B) Inflorescence biomass. (C) Reproductive allocation (the ratio of inflorescence biomass to total aboveground biomass). (D) Aerenchyma production (the ratio of stem aerenchyma area to total stem area, measured from stem cross sections), for which all standard errors < 0.012.

Tests of Hyp. 1 revealed that the two species differed in their trait values and responses to flooding (Table 1.1). Comparisons of flowering prior to the start of the experiment showed that *L. virgatum*’s 28% flowering rate did not differ statistically from *L. salicaria*’s 9% flowering rate (Χ^2^ = 2.564, P = 0.109). After adjusting for pre-treatment biomass via the covariate, aboveground biomass response to flooding also did not differ between species (Table 1.1, Fig. 3A). *Lythrum virgatum* did produce significantly more total inflorescence biomass, 30% more than *L. salicaria* (Table 1.1, Fig. 3B), and allocated a 76% higher proportion of its aboveground biomass to inflorescences (Table 1.1, Fig. 3C). Averaged across species, flooding decreased aboveground biomass by 29% (Fig. 3A, Table 1.1). Flooding decreased total inflorescence biomass by 40% more in *L. virgatum* than in *L. salicaria* (Fig. 3B; interaction, Table 1.1). *Lythrum virgatum* also decreased reproductive allocation over six times more strongly in response to flooding than did *L. salicaria,* which did not shift its allocation under flooding (Fig. 3C, Table 1.1). Every flooded plant produced stem aerenchymatous phellum, with *L. virgatum* producing 7% more aerenchyma per stem area than *L. salicaria* (Fig. 3D). These species differences in traits and fitness in response to flooding were independent of differences in overall size (total aboveground biomass covariate, Table 1.1).

**Table 1.** Flooding tolerance in *Lythrum virgatum* and *L. salicaria*, from full results of linear mixed effects models (Pinheiro *et al.* 2020) testing Hypothesis (Hyp.) 1, that each species has comparable traits and flooding tolerances (Table 1.1), and Hyp. 2, that aerenchyma production increases fitness in both species when flooded (Table 1.2).

Trait	Predictor	*β*	*β* standard error	df_1_	df_2_	*χ* ^2^	*P*
**Table 1.1 (Hyp. 1)**							
Total aboveground biomass (g)	Species	−1.799	1.186	1	23	2.499	0.114
	**Flooding**	**−2.657**	**0.728**	**1**	**29**	**14.441**	**<0.001**
	Species × flooding	−0.159	1.450	1	29	0.013	0.909
	**Pre-treatment total biomass (covariate)**	**0.690**	**0.211**	**1**	**29**	**11.645**	**<0.001**
Inflorescence biomass (g)	**Species**	**1.134**	**0.120**	**1**	**23**	**98.264**	**<0.001**
	Flooding	−0.040	0.111	1	28	0.147	0.702
	**Species × flooding**	**−0.413**	**0.204**	**1**	**28**	**4.527**	**0.033**
	Pre-treatment inflorescence biomass (covariate)	0.008	0.192	1	28	0.002	0.963
	**Total aboveground biomass (covariate)**	**−0.413**	**0.204**	**1**	**28**	**123.810**	**<0.001**
Reproductive allocation (inflorescence/total aboveground biomass)	**Species**	**0.068**	**0.017**	**1**	**23**	**16.097**	**<0.001**
	Flooding	−0.013	0.008	1	28	2.824	0.093
	**Species × flooding**	**−0.033**	**0.012**	**1**	**28**	**9.096**	**0.004**
	Pre-treatment repro. allocation (covariate)	0.138	0.110	1	28	1.758	0.185
	**Total aboveground biomass (covariate)**	**0.004**	**0.001**	**1**	**28**	**9.938**	**0.002**
Aerenchyma production (stem aerenchyma/total stem area)	**Species**	**0.041**	**0.006**	**1**	**23**	**50.722**	**<0.001**
	**Flooding**	**0.882**	**0.005**	**1**	**28**	**29638.373**	**<0.001**
	**Species × flooding**	**0.094**	**0.010**	**1**	**28**	**94.528**	**<0.001**
	Total aboveground biomass (covariate)	−0.001	0.001	1	28	2.575	0.109
**Table 1.2 (Hyp. 2)**							
Inflorescence biomass (g)	Aerenchyma production	0.835	1.508	1	19	0.366	0.545
	**Species**	**0.731**	**0.121**	**1**	**19**	**43.856**	**<0.001**
	Pre-treatment inflorescence biomass (covariate)	0.242	0.302	1	19	0.766	0.382
	**Total aboveground biomass (covariate)**	**0.101**	**0.018**	**1**	**19**	**37.634**	**<0.001**
Reproductive allocation (inflorescence/total aboveground biomass)	Aerenchyma production	−0.232	0.205	1	19	1.524	0.217
	Species	0.011	0.014	1	19	0.799	0.371
	Pre-treatment repro. allocation (covariate)	0.132	0.090	1	19	2.567	0.109
	**Total aboveground biomass (covariate)**	**−0.010**	**0.003**	**1**	**19**	**10.833**	**0.001**

Notes: Interpretation of species β coefficients are such that the mean for *L. virgatum* is compared to the mean for *L. salicaria*. For the flooding term, the mean for flooded treatment is compared to the mean for the non-flooded treatment. For the interaction term, the mean effect of flooding on *L. virgatum* is compared to the mean effect for *L. salicaria*. Model *P*-values are from type III Wald’s *χ*^2^ tests (Fox and Weisberg 2019). Bold denotes significance (*P* < 0.05).

**Figure 4.**
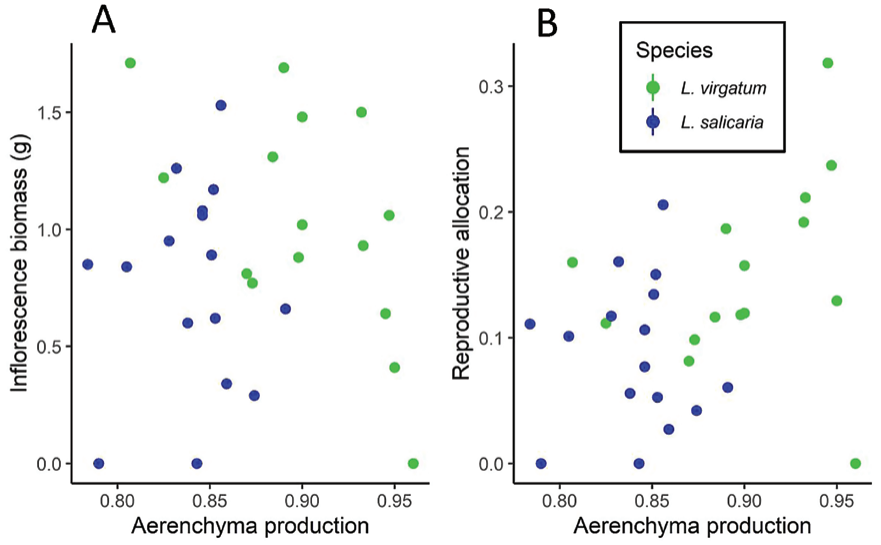
For flooded individuals of *Lythrum virgatum* and *L. salicaria*, relationships between aerenchyma production (the ratio of stem aerenchyma area to total stem area, measured from stem cross sections) and two fitness metrics. (A) Inflorescence biomass. (B) Reproductive allocation (the ratio of inflorescence biomass to total aboveground biomass).

Tests of Hyp. 2 allowed us to determine whether propensity to produce aerenchyma was adaptive (Table 1.2). We found no relationship between aerenchyma production and total fitness (inflorescence biomass, Table 1.2, Fig. 4A) or reproductive allocation (Table 1.2, Fig. 4B). Any apparent relationships were explained by differences in overall size (total aboveground biomass covariate, Table 1.2).

## DISCUSSION

We sought to investigate whether the little-studied ornamental species *L. virgatum* is similar to or different from *L. salicaria* in ecologically meaningful traits and in response to flooded conditions. We revealed similarities as well as important differences that challenge the assumption that these two taxa are indistinguishable (Rendall 1989; Anderson and Ascher 1993; Strefeler *et al*. 1996 b; Wilson *et al.* 2004; Anderson 2019). Our findings are relevant in the applied context of *L. salicaria* invasions. *L. virgatum* may be an underappreciated store of adaptive trait diversity.

### Higher fitness in L. virgatum than L. salicaria

One of our main findings was that *L. virgatum* had higher fitness than *L. salicaria* based on inflorescence biomass and reproductive allocation. This finding does not support the Hyp. 1 prediction of overall similarity between the two species. Fitness differences between the two species might be explained by constraints between flowering time and overall size. Flowering time is a genetically-based trait that governs adaptive responses in *L. salicaria* (Olsson 2004; Colautti and Barrett 2011, 2013; Wu and Colautti 2022). Flowering time also experiences a tradeoff with size, where smaller *L. salicaria* genotypes flower earlier, and larger genotypes flower later, putting on more vegetative biomass before shifting their allocation to reproduction (Bastlová and Květ 2002; Olsson and Ågren 2002; Bastlová *et al.* 2004; Montague *et al.* 2008; Colautti *et al*. 2010a; Colautti and Barrett 2011, 2013; Middleton *et al.* 2019; Wu and Colautti 2022). Consistent with the flowering time-size tradeoff, we saw trends wherein *L. virgatum* plants were smaller than *L. salicaria* and had more individuals flowering at the start of the experiment (though these differences were not statistically significant). By the end of the experiment, the larger *L. salicaria* consistently allocated more of its aboveground biomass to vegetative growth while the smaller *L. virgatum* allocated more to reproduction. As a result, *L. virgatum* was more fit based on our fitness metrics under both flooded and non-flooded conditions. If given more days to grow, our *L. salicaria* might have reached total fitness comparable to *L. virgatum.* For consistency, we harvested all individuals at the same time, when the flooded individuals were starting to senesce but potentially before some non-flooded individuals had reached their full reproductive output. The timing of our experiment is however reflected in relevant real-world scenarios. At more northern latitudes, earlier frosts might cut the growing season short. Earlier flowering might allow *L. virgatum* to thrive in the north, while *L. salicaria* might be more suited to establishing in all but the most northerly parts of the range. In *L. salicaria,* the early-flowering strategy predominates in the north (Olsson and Ågren 2002; Bastlová *et al.* 2004; Montague *et al.* 2008; Colautti *et al*. 2010a) and can be adaptive (Shadel and Molofsky 2002; Colautti and Barrett 2013; Wu and Colautti 2022). At southern latitudes with longer growing seasons, the late-flowering strategy is more common (Olsson and Ågren 2002; Bastlová *et al.* 2004; Colautti *et al*. 2010a) and adaptive (Montague *et al.* 2008; Colautti and Barrett 2013; Middleton *et al.* 2019; Wu and Colautti 2022). Studies are needed to examine potential local adaptation and parse variation in flowering time strategies across these species. Variation at the within-species level should also be examined by separately examining multiple genotypes of each species.

Differences in flowering between *L. salicaria* and *L. virgatum* might be controlled by a simple mechanism: whole genome duplication. *Lythrum virgatum* is diploid (2x = 30; Graham and Cavalcanti 2001), and *L. salicaria* from the source locations we used are most likely tetraploids (4x = 60; Kubátová *et al.* 2008). Consistent with our observations for the likely-polyploid, later-flowering *L. salicaria*, polyploids can show slowed growth and delayed flowering (del Pozo and Ramirez-Parra 2015; Laport *et al.* 2016). If ploidy does constrain flowering time, *L. virgatum* might be able to flower earlier than any *L. salicaria* (except perhaps for rare diploid *L. salicaria*, Kubátová *et al.* 2008). Higher fitness in *L. virgatum* versus *L. salicaria* might be generalizable beyond our experimental conditions because polyploids commonly show decreased allocation to sexual reproduction (Levin 1975; Van Drunen and Husband 2018). It is also likely, however, that diverse genotypes of both species contribute more to trait variation than ploidy alone, encompassing much more diversity than was captured by the two sets of genotypes per species that we used.

We quantified fitness as inflorescence biomass and reproductive allocation. *Lythrum virgatum* fitness traits are previously unexamined, but *L. salicaria* researchers have used fitness proxies including seed set (Montague *et al.* 2008; Colautti *et al*. 2010a), fruit number (Colautti *et al*. 2010*a*, *b*; Colautti and Barrett 2013), flower number (Chun *et al.* 2007; Chun 2011), and inflorescence biomass (Bastlová and Květ 2002; Bastlová *et al.* 2004; Colautti and Barrett 2011; Middleton *et al.* 2019; this study). We intentionally did not allow individuals to create mature fruits and to set seeds, given their invasive status in the region where we conducted this study. Using floral biomass as our fitness metric assumes that having more flowers is adaptively important. Rather than reproduction, environmental factors might limit these long-lived perennials.

### *Different responses to flooding in* L. virgatum *and* L. salicaria

Flooding is one factor with the potential to impact *Lythrum* performance. In our experiment, flooding caused both species to decrease total biomass and inflorescence biomass. Some studies of *L. salicaria* have seen opposite effects of flooding stress, including increased allocation either to floral production or to aboveground vegetative biomass (Mal *et al.* 1997; Bastlová *et al.* 2004; Chun *et al.* 2007). Our differing results likely result from our use of a relatively intense duration and degree of inundation, near the upper tolerance recorded for *L. salicaria* in the wild (NCHRP 1996). This intense flooding stressed *L. virgatum* more than *L. salicaria,* evidenced by *L. virgatum*’s stronger decreases in both fitness metrics. The mechanism behind these responses could be differences in flowering time, the key trait that controls adaptive responses in *L. salicaria* (Olsson 2004; Colautti and Barrett 2011, 2013; Wu and Colautti 2022). The smaller, earlier-flowering *L. virgatum* might respond more strongly to stress than the larger, later-flowering *L. salicaria.* We did not measure whether flooding affected flowering time but did qualitatively observe other phenological effects: flooding resulted in earlier onset of senescence and, in one case, resulted in mortality. Although *L. virgatum* was more stressed by flooding, it still maintained higher fitness than *L. salicaria* in both conditions of our experiment. Under stress, which suppressed fitness of both species, any fitness differences that are present could be of particular adaptive importance—i.e., the early-flowering strategy might be more advantageous in the face of stress. Overall, these differential stress responses add another piece of evidence against the Hyp. 1 prediction of similarity between the two species.

That prediction was also challenged by our findings regarding another aspect of flood response, production of aerenchymatous phellum on the stem. Aerenchyma had been documented in *L. virgatum* (Schenck 1889) but had never been compared to *L. salicaria*. We found *L. virgatum* produced more aerenchyma than *L. salicaria* (irrespective of differences in aboveground biomass). Aerenchyma is a specialized tissue produced under inundation to maintain aeration (Stevens *et al.* 2002) and is presumably costly to create. That *L. virgatum* produced more aerenchyma further illustrates our flooded condition as more stressful to *L. virgatum* than *L. salicaria.* Amount of aerenchyma, however, did not correlate with fitness in our study (Hyp. 2), suggesting aerenchyma production was a homeostatic response, rather than being adaptive (bolstering fitness) or maladaptive (at the cost of fitness). Other researchers have likewise hypothesized that presence of aerenchyma has not conferred an adaptive advantage to *L. salicaria* over other aerenchyma-producing Lythraceae species (Lempe *et al.* 2001). However, aerenchyma might bolster fitness over longer durations of flooding than those we explored. Previous work in *L. salicaria* has shown that stem aerenchymatous phellum functions to maintain overall aboveground biomass, a benefit which increases over the duration of flooding (Stevens *et al.* 2002).

Longer or repeated inundations might also affect fitness through interactions with flowering time differences. When allowed to grow for longer, the later-flowering pattern of our *L. salicaria* might be more advantageous, and is predicted to be increasingly favored as climate change lengthens growing seasons (Colautti *et al.* 2017). Novel inundation regimes are also resulting from climate change and human land use (AghaKouchak *et al.* 2020). Some areas are experiencing more inundation, including the core of the invasive range in Eastern North America, which is showing higher flood frequencies (Mallakpour and Villarini 2015) and higher maximum daily stream flows (Do *et al.* 2017). These changes will make differential flood tolerance increasingly relevant for these species.

### *Habitat tolerances in* L. virgatum *and* L. salicaria

The finding that *L. virgatum* was more stressed by flooding than was *L. salicaria* suggests that, in nature, moisture gradients might structure habitat differentiation between the two species. Such effects would likely operate at a within-ecosystem scale because individual wetlands often span substantial moisture gradients. In the context of invasion, such stratification might indicate *L. virgatum* would preferentially naturalize into less inundated wetland zones than those occupied by *L. salicaria.* Other environmental factors surely control establishment and persistence of *L. virgatum* in nature. For example, specialist beetles incur greater damage on early-flowering genotypes (Lehndal and Ågren 2015) and might prefer *L. virgatum*, especially because it is less hairy. Examining potential interactions with these beetles, which are widely used for biocontrol in the invasive range, should be prioritized (Colautti *et al.* 2017).

Although the two taxa show important differences, both are adapted to flooded environments and, when found in local proximity, hybridization may be a factor affecting plant habitat tolerances. Studies indicate that hybridization is not only possible (Anderson and Ascher 1993; Lindgren and Clay 1993; Ottenbreit and Staniforth 1994; Amon *et al.* 2007) but likely, meaning the unique trait variation we have documented for *L. virgatum* could introduce adaptive novelty into long-established *L. salicaria* invasions. Future studies should further examine hybridization in nature, although ploidy differences might make hybridization rare and thus difficult to detect. Climate change might provide more chances for hybridization by virtue of lengthened growing seasons, which would extend both flowering and pollinator activity (Colautti *et al.* 2017). Examining hybridization could start with observations of traits and genetics in natural populations in the introduced range. Genetics in this system have thus far only been studied with methods that have limited reproducibility or low resolution (Strefeler *et al*. 1996 a, b; Houghton-Thompson *et al.* 2005; Kubátová *et al.* 2008; Chun *et al.* 2009; Middleton *et al.* 2019; Jocienė *et al.* 2022), which—along with hybridization—leave *Lythrum* taxonomy largely unresolved. As genetic tools become increasingly accessible, biologists are creating a more generalized understanding rapid evolution in introduced species. This system, for which local adaptation in the invasive range has already been demonstrated, could provide an excellent model.

## Supporting Information

The following additional information is available in the online version of this article –

**Supporting Information 1.** The dataset of all measured traits and experimental design variables.

plad009_suppl_Supplementary_Data

## Data Availability

Data is available as Supporting Information. Data and R scripts are also deposited with the Dryad repository: https://doi.org/10.5061/dryad.dr7sqvb32.
